# Vitamin D Levels in Children with Adenotonsillar Hypertrophy and Otitis Media with Effusion

**Published:** 2017-01

**Authors:** Alimohamad Asghari, Zohreh Bagheri, Maryam Jalessi, Mohammad Mahdi Salem, Elahe Amini, Sahand GhalehBaghi, Sepideh Bakhti

**Affiliations:** 1 *Skull Base Research Center, ENT and Head & Neck Research Center, Hazrat Rasoul Akram Hospital, Iran University of Medical Sciences, Tehran, Iran.*; 2 *ENT and Head & Neck Research Center, Hazrat Rasoul Akram Hospital, Iran University of Medical Sciences, Tehran, Iran.*

**Keywords:** Adenoids, Child, Vitamin D, Otitis Media, Seasons

## Abstract

**Introduction:**

Vitamin D has been suggested to play a considerable role in the function of the immune system in various infectious, inflammatory, and autoimmune conditions. Otitis media with effusion (OME), defined as the presence of non-purulent fluid within the middle ear without signs or symptoms of suppurative otitis media, has a number of inflammatory predisposing factors. This study was designed to explore the association between vitamin D deficiency and OME.

**Materials and Methods:**

In this cross-sectional study, 74 children aged 2–7 years with an obstructive indication for adenotonsillectomy were included. Patients were divided into two groups based on the need for ventilation tube insertion for OME. Thirty-two children were enrolled in the OME group and 42 in the control group. The mean vitamin D level was compared between the two groups.

**Results:**

Mean vitamin D concentration in all patients was 11.96±5.85 ng/ml (9.79±4.36 ng/ml in the OME group and 13.61±6.33 ng/ml in the control group; P=0.003). There was also a significant difference in levels of vitamin D in patients referred in winter (9.0±2.94 ng/ml) compared with the summer (19.85±4.21 ng/ml; P=0.001). Data analyzed based on the season in which the patients were referred showed no significant difference between the OME and the control group.

**Conclusion:**

Although our results showed lower serum levels of vitamin D in OME patients, the difference was not significant when seasons were taken into consideration. Therefore, the season is an important confounding factor in any research related to vitamin D due to the effect of sun-induced vitamin D.

## Introduction

Vitamin D has been suggested to play an important role in improving the efficiency of the immune system. Proposed mechanisms of such action include the production of defensin, cathelicidin, and other antimicrobial peptides (1,2). Additionally, this vitamin improves the function of macrophages through increasing chemotaxis and phagocytosis (3). Although the relationship between vitamin D deficiency and infectious conditions was initially proposed in the context of tuberculosis, low levels of vitamin D are also shown to be highly associated with lower and upper respiratory tract infections (4–6). Furthermore, vitamin D has been suggested to have a role in some autoimmune diseases and inflammatory conditions such as allergy and asthma (7-9,10). Some investigations have demonstrated that vitamin D can affect the function of T-cells, including anti-proliferation effects on CD4^+^ T-cells and inhibitory effects on T-helper 1 and 2 (Th1 and Th2)-associated cytokine production; e.g. interleukin 4 and 13 (IL 4 and IL13) (8–11).

Otitis media with effusion (OME) is defined as an accumulation of non-purulent fluid in the middle ear without any signs or symptoms of acute infection (12,13). It is more prevalent between school and preschool children, with an incidence of up to 80% (14). As the symptoms of OME are less prominent compared with other ear diseases, diagnosis is often delayed which can lead to hearing impairment and consequent learning problems in children (15).

 The exact reason for the development of OME in children is not clearly understood. Inflammation, infection, and dysfunction of the pneumatic cells of the middle ear are the most important and common causes of OME (12). 

Although recurrent acute otitis media has been suggested as having an infectious cause, passive smoking, low socioeconomic status, day-care attendance, Eustachian-tube dysfunction, allergy, and imbalance of immune system have also been shown to be related to this condition (12,16). As vitamin D deficiency is thought to act as an aggravator of infectious and inflammatory conditions, we attempted to reassess the issue in this study by measuring the serum 25-OH vitamin D levels in children with and without OME.

## Materials and Methods

In this cross-sectional study, 74 children aged 2–7 years with an obstructive indication for adenotonsillectomy from September 2013 to March 2014 were included. The indication for surgery was obstructive sleep apnea diagnosed by a combination of signs and symptoms including habitual snoring, witnessed apnea, restless sleep, daytime symptoms of somnolence, behavior changes, or poor cognitive performance, and adenotonsillar hypertrophy (17). Patients were divided into case and control groups based on the need for ventilation tube (VT) insertion. The indication for VT insertion was defined by having OME (haziness of the tympanic membrane on otoscopic examination and tympanometry type B [flat 18]) that was unresponsive to medical management with antibiotics and antihistamines, according to the clinical practice guidelines for OME (18). No patients had received steroids for the treatment of their condition. This group of patients underwent VT insertion and adenotonsillectomy simultaneously, while children in the control group, with a normal tympanograms (type A (19)), underwent an adenotonsillectomy only. Accordingly, 32 children were enrolled in the OME group and 42 cases in the control group.

Patients who had a known chronic or systemic disease including rickets, renal impairment, mucociliary dysfunction, immune deficiency, or history of cleft palate or lip surgery were excluded from the study. The study was approved by the ethics committee of the ENT Head and Neck Research Center, and informed consent was taken from the parents.

*Laboratory Methods*

Serum levels of 25-OH vitamin D were measured in all participants. A 5-mL blood sample was obtained from each patient in the operating room. The serum level of 25-OH vitamin D was measured using the enzyme-linked immunosorbent assay (ELISA) method via spectrophotometry with Diaplas Kit (ELISA reader, USA). The level of vitamin D reported using this kit (nmol/L) was then converted to ng/ml by dividing the reported number by 2.5. The following classification of 25-OH vitamin D status was used: deﬁcient (<10 ng/ml), insufﬁcient (≥10 and <20 ng/ml), normal level (≥20 and ≤100 ng/ml), and toxic level (>100 ng/ml) (20).

*Statistical analyses*

The data were analyzed using SPSS v.22 software (Chicago, IL, USA). For descriptive analysis, the quantitative variables were reported by means and standard deviations, and qualitative data by frequency and percentage. An independent sample *t*-test and Mann Whitney U-test were used to compare the mean serum level of vitamin D between the OME and control groups as well as between genders and seasons in which the surgery was performed. In all analytical procedures, P<0.05 was considered the statistically significant level.

## Results

Seventy-four children with a mean age of 5.45±1.41 years were enrolled in this study. The average age of the OME and control groups was 5.44±1.54 and 5.46±1.31 years, respectively. There were 12 girls and 20 boys in the OME group and 15 girls and 27 boys in the control group, with no significant difference in age and sex between the two groups.

The mean serum 25-OH vitamin D level in all patients was 11.96±5.85 ng/ml, with values of 9.79±4.36 and 13.61±6.33 ng/ml in the OME and control groups, respectively (p=0.003). Overall, only 15 (20.3%) patients had a normal serum 25-OH vitamin D level, while the remaining patients (79.7%) had a value below the normal range. The serum 25-OH vitamin D levels in the OME and control groups are shown in Table 1. According to gender, the differences in 25-OH vitamin D levels between girls and boys overall and in each group were not significant.

**Table 1:** The level of serum vitamin D in patients with or without otitis media with effusion

**Levels**	**OME ** a ** Group**	**Without OME ** a ** Group**	
	**Frequency (Number)**	**Frequency (Number)**	
Severely deficient	0% (0)	9.5%(4)	
Deficient	71.9%(23)	26.2%(11)	
Insufficient	21.9%(7)	33.3% (14)	
Sufficient	6.2%(2)	31%(13)	

a OME = Otitis Media with Effusion

Fifty-four children (73%) underwent surgery and laboratory testing in the winter. Of these, 54% belonged to the OME group and 46% belonged to the control group. Of the 20 (27%) patients who had surgery in the summer, 15% belonged to the OME group and 85% to the control group. There was a statistically significant difference in levels of 25-OH vitamin D between patients referred in the winter (9.04±2.94 ng/ml) compared with the summer (19.85±4.21 ng/ml; p=0.001). Case and control data were compared according to the season that the patient underwent surgery. In winter, the mean 25-OH vitamin D level in the OME and control groups were 8.97±2.60 ng/ml and 9.11±3.33 ng/ml, respectively, with no statistically significant difference between groups. In the summer, the mean 25-OH vitamin D level in the case and control groups were 17.70±9.81 ng/ml and 20.23 ±2.83 ng/ml, respectively, also with no significant difference between groups.

## Discussion

The relationship between vitamin D deficiency and various infections is the subject of several studies and seems to be an ongoing field of interest. Many studies have been conducted to investigate the important role of vitamin D in immune system function (4–9). Vitamin D helps immature macrophages convert to mature ones. This conversion increases the phagocytosis, chemotaxis, and bactericidal activity of these cells (3,21-23). Therefore, vitamin D deficiency has been suggested to increase the possibility of infection.

In order to assess the role of vitamin D in acute otitis media, Cayir et al. (23) measured the serum level of vitamin D in 84 children who suffered from recurrent otitis media and compared it with 108 healthy children. The difference between the two groups was statistically significant. Additionally, they found that supplementary treatment with vitamin D could decrease the rate of recurrence of otitis media, and concluded that vitamin D deficiency could be a risk factor for upper respiratory tract infections such as otitis media (23). Marchisio et al. (24) performed a randomized controlled trial on 116 children with recurrent acute otitis media. Fifty-eight children received 1,000 IU/day vitamin D supplement and others were treated with placebo. The children who received vitamin D supplement had lower attack rates. Moreover, when the serum level of vitamin D was greater than 30 ng/ml, the risk of acute otitis media significantly decreased (24).

Whereas the relationship between vitamin D deficiency and acute otitis media is undeniable, there is an insufficient body of research to draw the same definitive conclusion for OME. Linday et al. (25) conducted a study on 16 children with a mean age of 3.7 years who underwent VT insertion, and reported that in 50% of these children, the serum level of vitamin D was below 20 ng/ml. However, this study did not demonstrate any significant relationship between the level of vitamin D and OME (25).

Our study demonstrated a meaningful difference in level of vitamin D in children with OME compared with children without OME. Although this finding shows a significant association, this does not indicate an etiological relationship, as the patients with OME may have a prolonged course of disease that may influence their nutritional status. Accordingly, it was shown that 90% of OME cases had surgery in the winter time, when overall serum level of vitamin D was significantly lower than in the summer time (9.04±2.94 ng/ml compared with 19.85±4.21 ng/ml). It is known that the main proportion of vitamin D is made in our skin by exposure to the sunlight (26). Some investigations have highlighted the role of seasonal variations in sun-induced vitamin D level and have presented a higher rate of mortality in some cancers in the winter and autumn when the serum level of vitamin D is low (27,28). Therefore, the season can act as a confounding factor. When the season is considered, our study was not able to show any difference between the two groups.

A few studies have shown the role of vitamin D deficiency in adenotonsillar hypertrophy (29–31). In one study, most of the children who underwent adenotonsillectomy had vitamin D deficiency, and the level of vitamin D inversely correlated with tonsillar size (30). In another *in vitro* study by Nunn et al. (31), vitamin D was shown to prevent the mitogenic-induced proliferation of tonsillar tissue.

Considering the level of vitamin D in our patients, 79.7 % were categorized as insufficient or deficient (vitamin D level less than 20 ng/ml). In contrast, a study by Ardestani (32) which was conducted in 513 cases from the normal population from a similar area and aged 6–7 years reported that only 3% had a vitamin D level less than 20 ng/ml. As all of our patients had adenotonsillar hypertrophy requiring surgery, this may indicate a relationship between low level of serum vitamin D and adenotonsillar hypertrophy. However, a second control group of normal children is needed to be able to confirm this suggestion.As season is a factor that has influence in both the level of vitamin D and the prevalence of some conditions, especially upper and lower respiratory system infections, it may affect the results in a more complicated way and thereby impact on any conclusion from these studies. Accordingly, a study focused on one season and conducted with larger numbers of patients would be useful to better show the exact additional role of vitamin D in the development of OME in patients with adenotonsillar hypertrophy.

## Conclusion

Although our results primarily showed a lower serum level of vitamin D in OME patients with adenotonsillar hypertrophy compared with patients with adenotonsillar hypertrophy only, when the confounding factor of season was considered, the difference was not significant. The importance of considering the season in any research related to the effect of vitamin D on various diseases should be emphasized.
